# Worries About COVID-19 and Adolescents' Mental Health and Life Satisfaction: The Role of Sociodemographics and Social Support

**DOI:** 10.3389/fped.2022.847434

**Published:** 2022-04-25

**Authors:** Rubén Rodríguez-Cano, Laura Cortés-García, Vidar S. Ulset, Tilmann von Soest

**Affiliations:** ^1^PROMENTA Research Centre, Department of Psychology, University of Oslo, Oslo, Norway; ^2^Norwegian Social Research, OsloMet – Oslo Metropolitan University, Oslo, Norway

**Keywords:** worry, COVID-19, mental health, life satisfaction, adolescents

## Abstract

Worries related to the COVID-19 pandemic are associated with mental health problems and reduced life satisfaction. However, the association between different types of worries about COVID-19 and adolescent mental health is unclear. Moreover, there is a lack of information about whether certain groups of adolescents are more vulnerable to the adverse effects of worries and how social support may moderate these effects. Adolescents (*N* = 12,686) completed a survey during the lockdown in spring 2020 in Oslo, Norway (37% response rate, 56.4% girls). The results showed that adolescent worries could be categorized into worries related to infection and those related to the general negative effects of the pandemic. Multivariate regression analyses showed that both types of worries were negatively related to positive affect and life satisfaction and positively related to depressive symptoms. Interaction analyses indicated that some associations with positive affect and depressive symptoms were stronger among adolescents with non-migrant backgrounds, higher family SES, and high reported levels of social support and physical contact during the pandemic. The findings suggest that COVID-19 worries may have negative effects on mental health and inform strategies to increase tailored psychological interventions to mitigate the effect of worry on adolescents' mental health and life satisfaction.

## Introduction

The SARS-CoV-2 pandemic has had a profound impact on worldwide health and economies (1). Adolescents may be particularly affected as they must not only cope with the unprecedented situation, but also with the significant transitions involved in this developmental stage (2). Moreover, adolescence is a susceptible time for developing mental health problems (3) and, thus, adolescents may be particularly vulnerable to experiencing mental health problems as a consequence of physical distancing (4) and restrictions in leisure activities (5). Therefore, knowledge about factors associated with mental health and life satisfaction is required to reduce the negative impact of COVID-19 on adolescents.

A cognitive factor that may increase vulnerability to mental health problems during the pandemic is COVID-19-related worries (6). Worry is defined as a set of maladaptive, future-oriented, repetitive, and catastrophic thoughts regarding the possibility of future negative events (7). Higher levels of COVID-19-related worries are negatively associated with adolescent mental health (8) and life satisfaction (9). The majority of studies have analyzed COVID-19 worries from an unidimensional perspective (i.e., “I worry a lot about the coronavirus-19”) (10); however, a multidimensional approach may provide more detailed information about the relationship between worry and adolescents mental health. For example, Taylor et al. (11) identified different aspects of COVID-19-related worries among US adults, such as fear of infection and socio-economic consequences. Furthermore, adolescent worries may differ from adult worries. Indeed, COVID-19-related financial worries are prevalent in adults but not children (12). Correspondingly, as adolescents have a lower risk for serious health consequences from COVID-19, their worries for their own health may be lower than for adults, whereas their worries about infecting others may be higher. Recent research indicated that the most common COVID-19-related worries among adolescents are related to the impact of the restrictions on academic careers (i.e., college admissions) and health effects of an infection (i.e., self-infection and family illness) (13). Other research also identified academic worries as one type of worry related to the COVID-19 pandemic (14). However, research is still limited regarding the different content of worries and their relation to adolescent mental health.

In addition to understanding COVID-19 worries and their associations with mental health, it is vital to identify subgroups of adolescents for whom worries may have particularly detrimental effects on mental health and life satisfaction. Preliminary evidence indicated that the pandemic has greater adverse effects on mental health among girls, younger adolescents, and adolescents with migrant backgrounds and low socio-economic status (SES) (15–18). A nationwide Norwegian study also confirmed that girls, younger adolescents, and adolescents with low parental education and from poor families showed greater adverse changes during the pandemic on mental health and social relationships than older adolescents (19). However, it is unknown whether these sociodemographic characteristics moderate the association between COVID-19 worries and adolescent mental health and life satisfaction.

Perceived social support is another factor that may moderate this association. Perceived social support is positively related to mental health and life satisfaction among adolescents (20) and also reduces distress resulting from stressful events (21). Interestingly, although social contact may reduce the negative association between COVID-19 and mental health (22), more in-person contact may also increase adolescents' worries about COVID-19 infections resulting from such contact. Furthermore, adolescents' online interaction with friends was found to be related to higher levels of mental health problems during the pandemic (17). Therefore, social support and the source of social contact may moderate the relationship between worry about COVID-19 and adolescent mental health and life satisfaction.

Taken together, the current study aims to better understand the relationship between COVID-19-related worries and adolescent mental health and life satisfaction, and to distinguish the role of sociodemographic variables (gender, age, migrant background, and SES), perceived social support (support from peers and family) and type of contact (physical and online) for these associations. We hypothesize that: (1) higher levels of COVID-19-related worries are associated with poorer mental health and lower life satisfaction; (2) this association remains when controlling for gender, age, migrant background, SES, social support, and social contact; (3) the association of COVID-19-related worries with mental health and life satisfaction is stronger among girls, younger adolescents, adolescents with a migrant background and adolescents with lower family SES. Since literature on this topic is limited, we do not predict a specific direction of interactions with social support and type of contact.

## Methods

### Procedure and Participants

Data were used from a large-scale population-based survey conducted between April 23 and May 8, 2020, in Oslo, the capital of Norway. At the time of assessment, all schools in Norway were closed due to the COVID-19 pandemic and students were attending digital schooling from home. The study was carried out by Norwegian Social Research (NOVA) at Oslo Metropolitan University. The school authorities in Oslo asked all public junior and senior high schools to participate in the study. Participants completed a digital questionnaire during a 30-min digital classroom session. From the eligible students, 37% (*N* = 12,686) participated. Previous publications have compared socio-demographic characteristic of our sample to population data about adolescents in Oslo from Statistics Norway (23). Results showed that the proportion of girls in the present sample was higher than in the population (56% vs. 50%), whereas the proportion of adolescents with migrant background was lower (30% vs. 37%). The study was anonymous and exempt from approval by the Regional Committee of Medical and Health Research Ethics. Students received written information outlining the study objectives and stating that the study was anonymous, and participation was voluntary. Parents were informed in advance about the study.

### Measures

#### COVID-19-Related Worries

Similar to studies that analyzed COVID-19 worries from a bidimensional perspective among adults (i.e., worry about dangerousness of COVID-19 and about socio-economic impact) (11), COVID-19-related worries were conceptualized as worries related to infection (3-items: worry about own illness, infecting others, and illness of family members or friends; Cronbach's alpha = 0.73) and worries about academic and economic consequences of COVID-19 (3-items: worries about family economic situation, impact on school grades, and the country's economy; Cronbach's alpha = 0.56). Response options ranged from *not worried at all* (1) to *very worried* (4). We conducted confirmatory factor analyses to analyze the latent structure of the items. Firstly, a one-factor solution was modeled with all items loading on one factor; however, this model did not show satisfactory fit (χ^2^(9) = 2013.99, *p* < 0.001; CFI = 0.85; RMSEA = 0.137; SRMR = 0.068). Following this, a two-factor solution was modeled with correlated latent factors. The three items related to worries about COVID-19 infection loaded onto one factor, whereas the remaining three items about academic and economic consequences loaded onto a second factor. This model showed satisfactory fit (χ^2^(8) = 298.07, *p* < 0.001; CFI = 0.98; RMSEA = 0.055; SRMR = 0.025). The two latent worry factors were positively correlated *(r* = 0.52, *p* < 0.001).

#### Mental Health and Life Satisfaction

Mental health was assessed with two instruments regarding positive affect and depressive symptoms. Positive affect was assessed using a 6-item scale about the frequency of positive feelings during the last seven days (e.g., “felt happy, engaged, energetic”; Cronbach's alpha = 0.87). Response options ranged from *not at all* (1) to *all the time* (5). Depressive symptoms were measured using a 6-item version of the Hopkin Symptom Checklist (24, 25). Response options ranged from *not affected* (1) to *extremely affected* (4). Internal consistency was high (Cronbach's alpha = 0.87). Life satisfaction was measured using the Cantril's ladder (26), with response options ranging from *worst possible life* (0) to *best possible life* (10).

#### Sociodemographics

Gender, age (i.e., school grade) and migrant background (i.e., at least one parent born abroad) were assessed by self-report. Family socio-economic status was assessed by a composite score comprising the number of books at home, level of education of parents, and four items from Family Affluence Scale II (27), which include frequency of traveling for family holidays in the previous year, number of computers and cars in the family, and the participant having an individual room at home. Higher scores indicated higher SES.

#### Social Support

Peer social support was measured using one item: “Do you have at least one friend who you completely trust and to whom you can reveal everything?” Response options were *yes, certainly* (4), *yes, I think*, (3) *I don't think so* (2), *I don't have anyone I would call a friend, these days* (1). Family social support was assessed using three items from a short version of the Parental Bonding Instrument (Cronbach's alpha = 0.68) (28), with higher scores indicating higher social support.

#### Physical and Online Contact

Physical and online contact were assessed using two questions: “How many of the previous 7 days have you been physically together with friends or a boyfriend/girlfriend?,” and “How many of the previous 7 days have you been in contact with friends or a boyfriend/girlfriend via the Internet or a mobile phone?.” Higher scores indicated higher levels of contact.

### Data Analyses

Descriptive statistics and correlation analyses were performed. Linear regression analyses were used to examine the association between worries about COVID-19 and mental health and life satisfaction. Analyses were presented with and without controlling for covariates. Following this, moderator analyses were conducted to assess whether socio-demographic factors and social support moderated the association between COVID-19-related worries and mental health and life satisfaction. When significant moderation effects were identified, simple-slope analyses were conducted at ±1 standard deviation of the moderator means. For categorical moderators (i.e., gender and migrant background), simple slopes were run for each group. Simple-slope analyses detected at which level of the moderators (i.e., socio-demographic and social support) predictor variables (i.e., worries about COVID-19) were related to mental health and life satisfaction outcomes (29). Analyses were conducted with R (v.4.0.3), using packages *psych* (v.2.0.12) (30), *interactions* (v.1.1.3) (31), and *lavaan* (v.0.6–7) (32). A significance level of *p* < 0.01 was used.

## Results

### Descriptive Statistics and Correlations

At the item level, adolescents worried more about infecting others (*M* = 2.90; *SD* = 0.93) and their families or friends getting infected (*M* = 2.54; *SD* = 0.96) than about being infected themselves (*M* = 1.69; *SD* = 0.76). Moreover, worries about the impact of the pandemic on their academic grades (*M* = 2.39; *SD* = 0.99) and the economy of the country (*M* = 2.20; *SD* = 0.89) were more common than worries about the family's financial situation (*M* = 1.74; *SD* = 0.90). Overall, adolescents showed a moderate level for worry about COVID-19 infection (*M* = 2.38; *SD* = 0.72) and academic and economic consequences of the pandemic (*M* = 2.11; *SD* = 0.68).

Descriptive statistics are presented in Table 1. Both types of worries were positively associated with being female, higher age, and migrant background, and negatively associated with family SES. Higher social support from peers was negatively related to worries about the academic and economic consequences of COVID-19, but not to worries about COVID-19 infection. Interestingly, family social support showed a small, positive relation to worries about infection, but was negatively related to worries about academic and economic consequences. Physical contact showed negative associations with both types of worry, but online contact showed a significant, albeit small, positive association only with worries about consequences.

**Table 1:** Means, standard deviations and correlations between all study variables (*N* = 12,686).

**Variable**	**M**	**SD**	**Range**	**1**	**2**	**3**	**4**	**5**	**6**	**7**	**8**	**9**	**10**	**11**	**12**
1. Worry infection	2.38	0.72	[1,4]												
2. Worry academic & economic	2.11	0.68	[1,4]	0.36^**^											
3. Positive affect	3.16	0.79	[1,5]	−0.05^**^	−0.25^**^										
4. Depressive symptoms	2.17	0.74	[1,4]	0.19^**^	0.39^**^	−0.64^**^									
5. Life satisfaction	6.08	2.00	[0,10]	−0.07^**^	−0.25^**^	0.60^**^	−0.54^**^								
6. Female gender, %	56.4	-	-	0.23^**^	0.15^**^	−0.15^**^	0.22^**^	−0.10^**^							
7. Age	3.01	1.61	[1,6]	0.05^**^	0.18^**^	−0.14^**^	0.18^**^	−0.11^**^	0.03^**^						
8. Migrant background, %	30.6	-	-	0.16^**^	0.18^**^	0.04^**^	−0.02^*^	0.03^**^	0.02^**^	−0.01					
9. Family SES	3.00	1.42	[1,5]	−0.13^**^	−0.18^**^	0.03^**^	−0.02^*^	0.02^*^	0.01	−0.00	−0.50^**^				
10. Social support peers	3.49	0.78	[1,4]	0.02^*^	−0.06^**^	0.21^**^	−0.15^**^	0.15^**^	0.06^**^	0.03^**^	−0.07^**^	0.08^**^			
11. Social support family	3.24	0.65	[1,4]	0.05^**^	−0.14^**^	0.37^**^	−0.34^**^	0.28^**^	0.04^**^	−0.03^**^	−0.07^**^	0.12^**^	0.21^**^		
12. Physical contact	2.43	1.13	[1,5]	−0.07^**^	−0.08^**^	0.11^**^	−0.05^**^	0.06^**^	−0.03^**^	−0.01	−0.26^**^	0.19^**^	0.21^**^	0.04^**^	
13. Online contact	4.19	1.11	[1,5]	0.03^**^	−0.01	0.08^**^	−0.00	0.04^**^	0.07^**^	0.06^**^	−0.10^**^	0.08^**^	0.28^**^	0.09^**^	0.28^**^

* *p < 0.01;*

** *p < 0.001*.

*M, mean; SD, standard deviation; Age, from grade 1 (13 years-old) to 6 (18 years-old)*.

### Worry About COVID-19 Infection

Regression results for worries about infection are displayed in Table 2. For models including positive affect, worry about COVID-19 infection predicted negatively positive affect. The association remained significant when adjusting for covariates. Adjusted analyses also showed that being male, being younger, having a migrant background, support from peers and family, and physical contact with friends predicted higher positive affect. Models that included the interaction terms of worry about COVID-19 infection and moderators showed significant moderation effects by migrant background, family SES, social support from peers, and physical contact. Simple-slope analyses indicated that worry about infection predicted significantly positive affect only among those adolescents with a non-migrant background (*B* = −0.09, SE = 0.01, *p* < 0.001), higher family SES (*B* = −0.10, SE = 0.01, *p* < 0.001), more social support from peers (*B* = −0.08, SE = 0.01, *p* < 0.001), and more physical contact with friends (*B* = −0.08, SE = 0.01, *p* < 0.001), compared to adolescents with a migrant background (*B* = −0.01, SE = 0.02, *p* = 0.60), lower family SES (*B* = −0.01, SE = 0.01, *p* = 0.35), less social support from peers (*B* = −0.04, SE = 0.01, *p* = 0.01) and less physical contact with friends (*B* = −0.01, SE = 0.01, *p* = 0.29).

**Table 2:** Regression analyses for the association between worries about COVID-19 infection on adolescents' mental health and life satisfaction.

**Model**		**Positive affect**			**Depressive symptoms**			**Life satisfaction**		
		**B**	**SE**	***t***	**B**	**SE**	***t***	**B**	**SE**	***t***
1^a^	Worry	−0.053	0.010	−5.385^**^	0.191	0.010	19.745^**^	−0.066	0.009	−6.846^**^
2^b^	Worry	−0.042	0.009	−4.430^**^	0.171	0.009	18.428^**^	−0.061	0.010	−6.227^**^
	Female gender	−0.326	0.019	−17.437^**^	0.394	0.019	21.300^**^	−0.208	0.020	−10.666^**^
	Age	−0.123	0.009	−13.777^**^	0.153	0.009	17.342^**^	−0.098	0.009	−10.534^**^
	Migrant background	0.248	0.023	10.655^**^	−0.178	0.023	−7.724^**^	0.165	0.024	6.836^**^
	Family SES	0.007	0.010	0.683	0.016	0.010	1.578	0.002	0.011	0.206
	Social support peers	0.131	0.010	13.508^**^	−0.101	0.010	−10.524^**^	0.108	0.010	10.712^**^
	Social support family	0.348	0.009	37.783^**^	−0.338	0.009	−37.047^**^	0.260	0.010	27.061^**^
	Physical contact	0.089	0.010	9.194^**^	−0.026	0.010	−2.694^*^	0.052	0.010	5.096^**^
	Online contact	0.017	0.010	1.807	0.030	0.010	3.178^*^	−0.006	0.010	−0.557
3^c^	Worry × Female gender	0.003	0.020	0.134	−0.014	0.020	−0.704	−0.006	0.020	−0.292
	Worry × Age	−0.014	0.010	−1.485	0.021	0.010	2.196	0.001	0.010	0.051
	Worry × Foreign origin	0.079	0.021	3.747^**^	−0.098	0.021	−4.710^**^	0.028	0.021	1.375
	Worry × Family SES	−0.042	0.010	−4.308^**^	0.033	0.010	3.401^*^	−0.006	0.010	−0.642
	Worry × Social support peers	−0.024	0.009	−2.589	0.031	0.009	3.388^*^	−0.014	0.009	−1.490
	Worry × Social support family	−0.007	0.009	−0.778	0.017	0.009	1.971	−0.012	0.009	−1.353
	Worry × Physical contact	−0.032	0.010	−3.322^**^	0.010	0.010	1.009	−0.013	0.010	−1.407
	Worry × Online contact	−0.019	0.009	−2.029	0.021	0.009	2.266	−0.009	0.009	−1.018

* *p < 0.01:*

** *p < 0.001*.

a *Unadjusted for covariates*.

b *Adjusted for all covariates*.

c *Adjusted for interaction main effects*.

For models with depressive symptoms as the outcome, higher levels of worry about COVID-19 infection predicted higher levels of depressive symptoms, both with and without adjustment for covariates. Being female, being older, having a non-migrant background, having more social support from peers and family, and having less physical contact also predicted higher levels of depressive symptoms. Models with interaction terms indicated a moderation effect of migrant background, family SES, and social support from peers. Specifically, the relationship between worry about COVID-19 infection and depressive symptoms was stronger among adolescents with a non-migrant background (*B* = 0.23, SE = 0.01, *p* < 0.001), higher family SES (*B* = 0.23, SE = 0.01, *p* < 0.001), and high levels of social support from peers (*B* = 0.23, SE = 0.01, *p* < 0.001), compared to adolescents with a migrant background (*B* = 0.14, SE = 0.02, *p* < 0.001), lower family SES (*B* = 0.16, SE = 0.01, *p* < 0.001), and less social support from peers (*B* = 0.16, SE = 0.01, *p* < 0.001).

Finally, when life satisfaction was the outcome, high levels of worry about COVID-19 infection predicted low life satisfaction in models both with and without covariate adjustment. Similar to positive affect, being male, being younger, and having migrant background predicted higher levels of life satisfaction. Furthermore, social support from peers and family, and physical contact with friends predicted life satisfaction. Results from moderation analyses showed no statistically significant interaction effects for any of the potential moderators.

### Worry About COVID-19's Academic and Economic Consequences

Regression models for worry about COVID-19's academic and economic consequences are presented in Table 3. Regarding positive affect models, worries about the academic and economic consequences were negatively related to positive affect both with and without covariates. There were similar associations between the covariates and positive affect as observed in the models with worry about COVID-19 infection. Moderation analyses did not show any significant interactions.

**Table 3:** Regression analyses for the association between worries about COVID-19's academic and economic consequences on adolescents' mental health and life satisfaction.

**Model**		**Positive affect**			**Depressive symptoms**			**Life satisfaction**		
		**B**	**SE**	***t***	**B**	**SE**	***t***	**B**	**SE**	***t***
1^a^	Worry	−0.251	0.010	−26.266^**^	0.390	0.009	42.875^**^	−0.246	0.009	−26.211^**^
2^b^	Worry	−0.181	0.009	−19.343^**^	0.319	0.009	35.376^**^	−0.204	0.010	−20.991^**^
	Female gender	−0.293	0.018	−16.111^**^	0.376	0.018	21.471^**^	−0.175	0.019	−9.303^**^
	Age	−0.095	0.009	−10.665^**^	0.108	0.009	12.556	−0.069	0.009	−7.370^**^
	Migrant background	0.291	0.023	12.693^**^	−0.226	0.022	−10.205^**^	0.207	0.024	8.706^**^
	Family SES	−0.007	0.010	−0.688	0.033	0.010	3.302^**^	−0.015	0.011	−1.419
	Social support peers	0.125	0.010	13.012^**^	−0.091	0.009	−9.833^**^	0.103	0.010	10.430^**^
	Social support family	0.325	0.009	35.501^**^	−0.292	0.009	−33.125^**^	0.232	0.010	24.469^**^
	Physical contact	0.087	0.010	9.058^**^	−0.027	0.009	−2.891^*^	0.050	0.010	4.995^**^
	Online contact	0.020	0.010	2.102	0.029	0.009	3.143^*^	−0.004	0.010	−0.403
3^c^	Worry × Female gender	0.006	0.020	0.316	0.025	0.019	1.332	0.007	0.019	0.374
	Worry × Age	−0.021	0.009	−2.198	0.020	0.009	2.183	−0.003	0.009	−0.268
	Worry × Foreign origin	0.019	0.020	0.945	−0.066	0.019	−3.419^**^	−0.026	0.020	−1.312
	Worry × Family SES	−0.024	0.010	−2.456	0.021	0.009	2.327	0.012	0.010	1.282
	Worry × Social support peers	−0.017	0.009	−1.899	0.028	0.009	3.299^*^	−0.018	0.009	−2.078
	Worry × Social support family	0.004	0.009	0.406	−0.007	0.008	−0.881	0.012	0.009	1.371
	Worry × Physical contact	−0.019	0.009	−1.992	0.017	0.009	1.882	0.003	0.009	0.367
	Worry × Online contact	−0.005	0.009	−0.567	0.006	0.009	0.649	0.006	0.009	0.693

* *p < 0.01:*

** *p < 0.001*.

a *Unadjusted for covariates*.

b *Adjusted for all covariates*.

c *Adjusted for interaction main effects*.

For models including depressive symptoms as the outcome, worries about COVID-19's academic and economic consequences predicted higher levels of depressive symptoms in both unadjusted and adjusted models. Covariates had similar associations with depressive symptoms as with worry about COVID-19 infection, but higher levels of online contact also predicted more depressive symptoms. Interaction analyses demonstrated that the relationship between worries about COVID-19's academic and economic consequences and depressive symptoms was stronger among adolescents with a non-migrant background (*B* = 0.43, SE = 0.01, *p* < 0.001) and with higher social support from peers (*B* = 0.41, SE = 0.01, *p* < 0.001), compared to adolescents with a migrant background (*B* = 0.37, SE = 0.02, *p* < 0.001) and lower support from peers (*B* = 0.37, SE = 0.01, *p* < 0.001).

For the model including life satisfaction, greater worries about COVID-19's academic and economic consequences predicted lower life satisfaction in both unadjusted and adjusted models. Covariates had similar relations to life satisfaction as in models investigating worry about COVID-19 infection. Results from moderation analyses showed no significant interaction effects.

## Discussion

This study analyzed the relationships between worries about the COVID-19 pandemic and adolescent mental health and life satisfaction and assessed whether these relationships were moderated by socio-demographic variables and social support. Results indicated that COVID-19 worries comprised two main dimensions: worries about COVID-19 infection and worries about COVID-19's academic and economic consequences. Findings also revealed that both types of worries predicted poor mental health (i.e., lower positive affect and higher depressive symptoms) and life satisfaction, even when controlling for covariates. Finally, our findings suggested that the relationships between worries and mental health were stronger among adolescents with non-migrant backgrounds, higher family SES, and high levels of social support or contact from peers. However, the relationships between both types of worry on life satisfaction were not moderated by any variables included in the models.

The bidimensional structure of worries about COVID-19 in adolescents, comprising worries about infection and worries about academic and economic effects, expands previous findings among US adults that the most relevant worries were the dangers of COVID-19 and the socio-economic impact of the pandemic (11). Our findings are also in line with a study with adolescents from India showing that the two most common worries were about the impact of COVID-19 on academic achievement (74% of the sample) and on health effects of an infection (41% of the sample) (13). In the present study, although adolescents reported similar, average levels of both types of worries (i.e., mean scores were around 2, on a 1–4 scale), the means varied on the item level. For example, regarding worries about infection, adolescents worried about the consequences of COVID-19 infection for others more than for themselves. Indeed, adolescents may perceive COVID-19 as less harmful for themselves than for older family members, in accordance with the lower COVID-19 morbidity and mortality among younger people (33). Moreover, adolescents were slightly more worried about COVID-19's academic and economic impact than about the family's financial situation. As such, in line with previous studies (13, 14), our results underline the importance of addressing the impact of both school-related stress and the economic situation on adolescents' worries.

As expected, higher levels of worries about COVID-19 were negatively related to both positive affect and life satisfaction, and positively depressive symptoms. These associations were also found when accounting for covariates. These findings are in accordance with previous studies indicating that COVID-19-related worries were associated with adolescent mental health and life satisfaction (8, 9). Additionally, our results extend previous literature by demonstrating that the relationship between adolescent worries and mental health should be considered from a dimensional perspective including various worries, not only about infection but also about the academic and economic consequences of the pandemic.

This study provides novel information about factors that moderate the relationship between worries, mental health, and life satisfaction among adolescents. Contrary to our expectations, the results did not show any moderation of the relationship between worries about COVID-19 and life satisfaction. In contrast, the associations between both types of worry and mental health were stronger among adolescents with non-migrant backgrounds and higher family SES. Our results might be partially explained by the fact that adolescents with a non-migrant background and high SES families may perceive the COVID-19 pandemic restrictions as being more severe, as they may participate in organized leisure activities more often than other adolescents, and these types of activities were restricted during the pandemic (9). Another tentative explanation of our findings could be that stress and worry levels may have already been high among migrant and low SES adolescents and, thus, COVID-19 may not have changed their level of worry to the same degree as for adolescents from high SES families. Moreover, Norwegian statistics show that registered coronavirus infections were more prevalent among high socio-economic strata in the beginning of the pandemic, right before this study was conducted (34). Adolescents from high SES backgrounds may as such have had more experience of and knowledge about the adverse consequences of COVID-19 infections, which may have strengthened the association between COVID-19 worries and mental health outcomes in this group of adolescents. Future longitudinal studies that investigate the change in worries and their resulting impact on mental health during COVID-19 may help to better understand these relationships.

Regarding worries predicting levels of mental health, the association was stronger among adolescents with higher levels of social support from peers. Previous studies observed that social support mitigates the negative impacts of difficult life events (20). However, other cognitive mechanisms may explain the stronger association between worries and mental health among adolescents with more social support. For example, adolescents receiving high levels of social support may have more opportunities to engage in co-ruminative processes that maintain and increase their distress in uncertain times (35). As a result, these adolescents may share their concerns and fears related to COVID-19 to a greater degree with their peers, thus increasing the negative effect of these worries on their mental health. Interestingly, when types of social contact were analyzed, the negative association between worry about COVID-19 infection and mental health was stronger among those with higher levels of in-person contact. Indeed, it is possible that adolescents may worry about infecting others through in-person contact. These worries may change in the post-pandemic period; however, future studies should explore the longitudinal influences of in-person and online contact on adolescent mental health in order to develop better infection control strategies and, thus, reduce infection rates.

Strengths of this study include the use of a large, population-based sample of adolescents (*N* = 12,686) and multidimensional assessment of worries. However, the results should be interpreted in the context of some limitations. First, the cross-sectional design limits the potential to uncover causal relationships between the variables. Future studies should include longitudinal designs. Second, measures may be affected by the limitations of self-report questionnaires, such as social desirability bias, so future research would benefit from multimethod assessment. Also, the bidimensional structure of the measure of COVID-19 worries may be a result of the selection of the six items used to assess this construct in the present study. A more comprehensive instrument with a wider item selection of potential worries may have resulted in a more multifaceted measure. Third, we did not examine whether having experienced a coronavirus infection was related to COVID-19 worries and mental health outcomes, as the survey did not include items on infections. Such information would have provided valuable information about the psychological consequences of an infection and should be examined in future studies. Fourth, although confirmatory factor analyses showed adequate model fit for a two-factor solution for COVID-19 related worries, this study is the first to use this instrument to assess COVID-19 worries in adolescents. Future studies should explore if the bidimensional solution on worries stands. Fifth, the 37% rate response in this study and the underrepresentation of boys and adolescents with migrant background may to some degree influence the estimates of prevalence of COVID-19 worries and associations between COVID-19 worries and mental health outcomes. Finally, it is unclear whether the present study results are generalizable to adolescents from countries other than Norway with different welfare systems. Therefore, research examining the effects of worry about COVID-19 in other countries would be beneficial.

## Conclusions

The current study enhances knowledge about the relationship between different types of worries about COVID-19 and mental health and life satisfaction among adolescents. Adolescents with higher SES and higher perceived social support levels may be more vulnerable to the association between high levels of worry about COVID-19 and poorer mental health. Future studies should monitor specific worries during the COVID-19 pandemic to reduce the risk of the development of mental health problems related to the pandemic. In addition, this study helps to inform therapeutic and prevention strategies that aim to reduce adolescents' worries about the COVID-19 pandemic and its continuous influence on adolescent mental health and life satisfaction.

## Data Availability Statement

The data used in this study is available from the corresponding author upon reasonable request and with the permission of Norwegian Social Research (NOVA).

## Ethics Statement

The study was anonymous and exempt from approval by the Regional Committee of Medical and Health Research Ethics. Written informed consent from the participants' legal guardian/next of kin was not required to participate in this study in accordance with the national legislation and the institutional requirements.

## Author Contributions

RR-C, TvS, and VU: methodology. RR-C: formal analysis. RR-C and VU: data curation. RR-C and LC-G: writing—original draft preparation. All authors contributed in conceptualization, writing—review and editing, read, and agreed to the published version of the manuscript.

## Funding

This study was supported by grants from the Research Council of Norway (Project nos. 288083 and 300816).

## Conflict of Interest

The authors declare that the research was conducted in the absence of any commercial or financial relationships that could be construed as a potential conflict of interest.

## Publisher's Note

All claims expressed in this article are solely those of the authors and do not necessarily represent those of their affiliated organizations, or those of the publisher, the editors and the reviewers. Any product that may be evaluated in this article, or claim that may be made by its manufacturer, is not guaranteed or endorsed by the publisher.

## References

[1] World Health Organization. Coronavirus. (2021). Available online at: https://www.who.int/westernpacific/health-topics/coronavirus (accessed: Dec 29, 2021).

[2] Steinberg L. Cognitive and affective development in adolescence. Trends Cogn Sci. (2005) 9:69–74. 10.1016/j.tics.2004.12.005 15668099

[3] Casey B Jones RM Levita L Libby V Pattwell S Ruberry E. The storm and stress of adolescence: insights from human imaging and mouse genetics. Dev Psychobiol. (2010) 52:225–35. 10.1002/dev.20447 20222060 PMC2850961

[4] Buzzi C Tucci M Ciprandi R Brambilla I Caimmi S Ciprandi G. The psycho-social effects of COVID-19 on Italian adolescents' attitudes and behaviors. Ital J Pediatr. (2020) 46:69. 10.1186/s13052-020-00833-4 32448323 PMC7245982

[5] Branquinho C Kelly C Arevalo LC Santos A de Matos MG. “Hey, we also have something to say”: a qualitative study of Portuguese adolescents' and young people's experiences under COVID-19. J Community Psychol. (2020). 48:2740–52. 10.1002/jcop.22453 33001455 PMC7537124

[6] Shepherd JM Fogle B Garey L Viana AG Zvolensky MJ. Worry about COVID-19 in relation to cognitive-affective smoking processes among daily adult combustible cigarette smokers. Cogn Behav Ther. (2021) 50:336–50. 10.1080/16506073.2020.1866657 33511905

[7] Davey G Wells A. Worry and Its Psychological Disorders: Theory, Assessment, and Treatment. Chichester; Hoboken, NJ: Wiley (2006). p. 410.

[8] Xie X Xue Q Zhou Y Zhu K Liu Q Zhang J. Mental health status among children in home confinement during the Coronavirus disease 2019 outbreak in Hubei Province, China. JAMA Pediatr. (2020) 174:898–900. 10.1001/jamapediatrics.2020.1619 32329784 PMC7182958

[9] von Soest T Bakken A Pedersen W Sletten MA. Life satisfaction among adolescents before and during the COVID-19 pandemic. Tidsskr Den Nor Legeforening. (2020) 140:1–10. 10.4045/tidsskr.20.0437 32602316

[10] Ahorsu DK Lin C-Y Imani V Saffari M Griffiths MD Pakpour AH. The fear of COVID-19 scale: development and initial validation. Int J Ment Health Addict. (2020) 1:1–9. 10.1007/s11469-020-00270-8 32226353 PMC7100496

[11] Taylor S Landry CA Paluszek MM Rachor GS Asmundson GJG. Worry, avoidance, and coping during the COVID-19 pandemic: a comprehensive network analysis. J Anxiety Disord. (2020) 76:102327. 10.1016/j.janxdis.2020.102327 33137601 PMC7585364

[12] Rothe J Buse J Uhlmann A Bluschke A Roessner V. Changes in emotions and worries during the Covid-19 pandemic: an online-survey with children and adults with and without mental health conditions. Child Adolesc Psychiatry Ment Health. (2021) 15:11. 10.1186/s13034-021-00363-9 33612122 PMC7897360

[13] Shukla M Pandey R Singh T Riddleston L Hutchinson T Kumari V. The effect of COVID-19 and related lockdown phases on young peoples' worries and emotions: novel data from India. Front Public Health. (2021) 9:645183. 10.3389/fpubh.2021.645183 34095054 PMC8172589

[14] Lessard LM Puhl RM. Adolescent academic worries amid COVID-19 and perspectives on pandemic-related changes in teacher and peer relations. Sch Psychol. (2021) 36:285–92. 10.1037/spq0000443 34292037

[15] Fegert JM Vitiello B Plener PL Clemens V. Challenges and burden of the Coronavirus 2019 (COVID-19) pandemic for child and adolescent mental health: a narrative review to highlight clinical and research needs in the acute phase and the long return to normality. Child Adolesc Psychiatry Ment Health. (2020) 14:20. 10.1186/s13034-020-00329-3 32419840 PMC7216870

[16] Dale R O'Rourke T Humer E Jesser A Plener PL Pieh C. Mental health of apprentices during the COVID-19 pandemic in Austria and the effect of gender, migration background, and work situation. Int J Environ Res Public Health. (2021) 18:8933. 10.3390/ijerph18178933 34501523 PMC8430826

[17] Magson NR Freeman JYA Rapee RM Richardson CE Oar EL Fardouly J. Risk and protective factors for prospective changes in adolescent mental health during the COVID-19 pandemic. J Youth Adolesc. (2021) 50:44–57. 10.1007/s10964-020-01332-9 33108542 PMC7590912

[18] Pierce M Hope H Ford T Hatch S Hotopf M John A. Mental health before and during the COVID-19 pandemic: a longitudinal probability sample survey of the UK population. Lancet Psychiatry. (2020) 7:883–92. 10.1016/S2215-0366(20)30308-4 32707037 PMC7373389

[19] von Soest T Kozák M Rodríguez-Cano R Fluit DH Cortés-García L Ulset VS. Adolescents' psychosocial well-being one year after the outbreak of the COVID-19 pandemic in Norway. Nat Hum Behav. (2022) 6:217–28. 10.1038/s41562-021-01255-w 35058644

[20] Rueger SY Malecki CK Pyun Y Aycock C Coyle S. A meta-analytic review of the association between perceived social support and depression in childhood and adolescence. Psychol Bull. (2016) 142:1017–67. 10.1037/bul0000058 27504934

[21] Camara M Bacigalupe G Padilla P. The role of social support in adolescents: are you helping me or stressing me out? Int J Adolesc Youth. (2017) 22:123–36. 10.1080/02673843.2013.875480

[22] Bekkhus M von Soest T Fredriksen E. Psykisk helse hos ungdommer under covid-19. Om ensomhet, venner og sosiale medier. Tidsskr Nor Psykologforening. (2020) 57:492−501.

[23] Statistics Norway. Statistisk sentralbyrå. SSB (2022). Available online at: https://www.ssb.no/ (accessed: Feb 23, 2022).

[24] Derogatis LR Lipman RS Rickels K Uhlenhuth EH Covi L. The Hopkins Symptom Checklist (HSCL). A measure of primary symptom dimensions. Mod Probl Pharmacopsychiatry. (1974) 7:79–110. 10.1159/000395070 4607278

[25] von Soest T Wichstrøm L. Secular trends in depressive symptoms among Norwegian adolescents from 1992 to 2010. J Abnorm Child Psychol. (2014) 42:403–15. 10.1007/s10802-013-9785-1 23888312

[26] Mazur J Szkultecka-Debek M Dzielska A Drozd M Małkowska-Szkutnik A. What does the Cantril Ladder measure in adolescence? Arch Med Sci AMS. (2018) 14:182–9. 10.5114/aoms.2016.60718 29379549 PMC5778415

[27] Currie C Molcho M Boyce W Holstein B Torsheim T Richter M. Researching health inequalities in adolescents: the development of the Health Behaviour in School-Aged Children (HBSC) family affluence scale. Soc Sci Med. (2008). 66:1429–36. 10.1016/j.socscimed.2007.11.024 18179852

[28] Parker G Tupling H Brown LB. A Parental Bonding Instrument. Br J Med Psychol. (1979) 52:1–10. 10.1111/j.2044-8341.1979.tb02487.x

[29] Hayes AF. Introduction to Mediation, Moderation, and Conditional Process Analysis: Second Edition: A Regression-Based Approach. NJ: Guilford Press (2018).

[30] Revelle W. Psych: Procedures for Psychological, Psychometric, Personality Research R Package Version 1.9.12. Northwestern University, Evanston. Available online at: https://CRAN.R-project.org/package=psych (2019).

[31] Long JA. Comprehensive, User-Friendly Toolkit for Probing Interactions. (2019). Available online at: https://interactions.jacob-long.com/ (accessed: May 21, 2021).

[32] Rosseel Y. lavaan: An R package for structural equation modeling. J Stat Softw. (2012) 48:1–36. 10.18637/jss.v048.i0225601849

[33] CDC. COVID-19 and Your Health. Centers for Disease Control and Prevention (2020). Available online at: https://www.cdc.gov/coronavirus/2019-ncov/daily-life-coping/parental-resource-kit/adolescence.html (accessed: Mar 31, 2021).

[34] Kraft KB Elgersma IH Labberton AS Indseth T Godøy A. COVID-19: Persons tested, confirmed cases and associated hospitalizations by education and income. Norwegian Institute of Public Health Division for Health Services; 2021. p. 20

[35] Rose AJ Glick GC Smith RL Schwartz-Mette RA Borowski SK. Co-rumination exacerbates stress generation among adolescents with depressive symptoms. J Abnorm Child Psychol. (2017) 45:985–95. 10.1007/s10802-016-0205-1 27624335 PMC5350052

